# Threshold of phenylacetylglutamine changes: exponential growth between age and gut microbiota in stroke patients

**DOI:** 10.3389/fneur.2025.1576777

**Published:** 2025-05-21

**Authors:** Yang Liu, Min Chu, Delong Wang, Qian Li, Jixian Lin, Jing Zhao

**Affiliations:** ^1^Department of Neurology, Minhang Hospital, Fudan University, Shanghai, China; ^2^Institute of Science and Technology for Brain-Inspired Intelligence (ISTBI), Fudan University, Shanghai, China; ^3^Department of Geriatrics, Shanghai Geriatric Medical Center, Shanghai, China

**Keywords:** phenylacetylglutamine (PAGln), ischemic stroke, age, gut microbiota, thrombosis risk

## Abstract

**Importance:**

phenylacetylglutamine (PAGln), a gut microbiota-derived metabolite, is linked to increased platelet reactivity and thrombosis risk. However, its relationship with age, particularly the non-linear patterns in ischemic stroke patients, remains unclear.

**Objectives:**

To explore the non-linear relationship between age and plasma PAGln levels in ischemic stroke patients, focusing on identifying exponential growth trends and critical age thresholds.

**Design, setting, and participants:**

This single-center, prospective cohort study was conducted at the Department of Neurology, Minhang Hospital, Fudan University, from January 2022 to December 2023. A total of 121 patients with ischemic stroke were consecutively enrolled. Demographic information, lifestyle factors, stroke characteristics, and comorbidities were systematically collected. Plasma PAGln levels were measured using rapid resolution liquid chromatography–quadrupole time-of-flight mass spectrometry. Generalized additive models and smoothing curve fitting were applied to assess non-linear relationships between age and PAGln levels, with threshold effect analysis used to identify age breakpoints. Multivariable regression models were applied to adjust for confounders, and subgroup analyses tested the robustness of findings.

**Main outcomes and measures:**

Plasma PAGln levels and their association with age in ischemic stroke patients, evaluated through non-linear models and regression analysis.

**Results:**

Significant differences in PAGln levels were found across age quartiles (*P* = 0.004), rising from 186.87 ± 95.49 μmol/L in the youngest quartile (35–54 years) to 433.11 ± 474.03 μmol/L in the oldest quartile (69–87 years). A non-linear association between age and PAGln levels was identified (*P* = 0.0006). Smoothing curve fitting revealed an exponential increase in PAGln levels with age. A threshold effect analysis pinpointed a breakpoint at 71 years. Below this age, no significant association between age and PAGln was observed (*P* = 0.5394), while above 71, a significant exponential increase in PAGln levels was detected (*P* < 0.0001). Subgroup analyses confirmed consistent results across various patient characteristics, with no significant interactions.

**Conclusions and relevance:**

A non-linear exponential relationship exists between age and plasma PAGln levels in ischemic stroke patients, with a marked increase after 71 years. Elevated PAGln levels in elderly patients suggest significant metabolic dysregulation, potentially raising thrombosis risk. Monitoring PAGln levels in stroke patients over 71 years could provide valuable insights for personalized interventions to reduce thrombotic complications.

## Introduction

Stroke remains a major global cause of death and disability, presenting an ongoing challenge for public health (1, 2). As the population ages, the incidence of stroke is rising, especially among older adults, who show significantly increased rates of both first-time and recurrent strokes (3). Recently, growing attention has been paid to the role of gut microbiota in neurological diseases, primarily through the “gut-brain axis,” which affects nervous system health and development (4, 5).

Phenylacetylglutamine (PAGln) is a key metabolite produced by the gut microbiota, recently linked to increased thrombosis risk (6). In 2020, Nemet et al. demonstrated that PAGln enhances platelet reactivity by modulating adrenergic receptors, thereby increasing thrombosis risk (7). This finding suggests that PAGln plays a key role in regulating the circulatory system. Additionally, the production of PAGln depends on the metabolic activity of the gut microbiota, and it is also considered a crucial marker reflecting the health of the gut and the metabolic burden of the host (8, 9).

Age is an inevitable factor that significantly influences both stroke onset and prognosis (10). With increasing age, the body's metabolism slows, immune regulation becomes less efficient, and vascular elasticity decreases. Furthermore, the composition of the gut microbiota in elderly populations is altered, characterized by reduced diversity and an increased proportion of pathogenic bacteria (11, 12). As a gut-derived metabolite, PAGln levels may exhibit a complex pattern of changes influenced by age and the composition of the microbiota. However, studies investigating the levels of PAGln in stroke patients and its relationship with age remain relatively scarce, particularly regarding whether this relationship exhibits a specific non-linear pattern.

This study aims to explore the non-linear relationship between age and PAGln levels in stroke patients, with a specific focus on whether an exponential growth trend exists. We hypothesize that PAGln levels may not simply accumulate linearly with age but instead undergo significant changes at certain age thresholds, exhibiting a non-linear exponential growth pattern. Based on this hypothesis, we conducted an in-depth analysis of clinical data from stroke patients and applied smoothing curve fitting and threshold effect analysis to uncover the potential complex associations between age and PAGln.

## Methods

### Study design and population

This single-center, prospective cohort study investigates the association between gut microbiota-derived metabolites and age in ischemic stroke patients. From January 2022 to December 2023, we consecutively enrolled 121 inpatients diagnosed with ischemic stroke from the Department of Neurology at Minhang Hospital, affiliated with Fudan University. The inclusion criteria were: age ≥ 18 years, National Institutes of Health Stroke Scale (NIHSS) score ≤ 5 upon admission, symptom onset within 7 days, and ischemic stroke diagnosis confirmed by magnetic resonance imaging (MRI). Exclusion criteria were: severe infections, autoimmune diseases, malignancies, recent use of antibiotics or probiotics within the past 3 months, and severe cardiac, hepatic, or renal insufficiency, and known digestive system diseases (e.g., inflammatory bowel disease, chronic diarrhea, gastrointestinal tumors, or history of major gastrointestinal surgery).

This study was conducted in strict accordance with the principles of the Declaration of Helsinki and was approved by the Ethics Committee of Minhang Hospital, Fudan University. All participants provided written informed consent.

### Baseline data collection

Baseline data were systematically collected through standardized questionnaires and medical records, covering: Demographic information (age, sex, weight, height, and BMI); Lifestyle and behavioral characteristics (smoking history, alcohol consumption); Stroke characteristics (ischemic cerebral infarction history, subtype classified according to the TOAST criteria); Comorbidities (hypertension, diabetes, atrial fibrillation, lipid metabolism disorders, and other relevant conditions); and Stroke severity (assessed upon admission using the NIHSS score). To facilitate stratified baseline comparisons, age was divided into quartiles based on ascending order.

### Measurement of gut microbiota-derived metabolites

Fasting venous blood samples were collected from patients within 24 h of admission using EDTA anticoagulant tubes. Samples were centrifuged at 1,000 × g for 10 min to separate plasma, which was aliquoted and stored at −80°C until analysis.

### Metabolite measurement method

Rapid resolution liquid chromatography-quadrupole time-of-flight mass spectrometry (RRLC-QTOF/MS) was employed to quantify plasma levels of gut microbiota-derived metabolites, including phenylacetylglutamine (PAGln), trimethyllysine (TML), and trimethylamine N-oxide (TMAO). Isotopically labeled internal standards (d5-PAGln, d9-TML, and d9-TMAO) were utilized to enhance quantification accuracy and reliability.

### Instrumentation and conditions

Liquid chromatography was performed using an Agilent 1,260 series rapid resolution system. Mass spectrometry detection was carried out using an Agilent 6,530 series quadrupole time-of-flight mass spectrometer equipped with a dual electrospray ionization source. Plasma and quality control samples were prepared according to standard operating procedures, which included protein precipitation, centrifugation, and filtration. Metabolite concentrations were determined by comparing peak area ratios of target metabolites to internal standards.

### Statistical analysis

All statistical analyses were performed using R software (version 4.0.2). Statistical tests were two-sided, with *P* < 0.05 considered statistically significant. Continuous variables were summarized as mean ± standard deviation (Mean ± SD) for normally distributed data or as median and interquartile range (IQR) for non-normally distributed data. Categorical variables were expressed as frequencies and percentages. Generalized additive models (GAMs) were used to assess the non-linear relationship between age and PAGln levels, and smoothing curves were plotted to visualize this trend. A segmented regression model was employed to identify any potential threshold point between age and PAGln levels, using the least squares method to estimate the threshold and compare the rate of change in PAGln levels before and after this point. Multivariable regression models were used to adjust for potential confounders, including gender, BMI, smoking history, alcohol consumption, hypertension, and diabetes. Sensitivity analyses and interaction tests were performed to verify the robustness of the findings.

## Results

### Baseline characteristics and metabolite levels

Among the 121 stroke patients, a significant difference in gender distribution was observed across the age quartiles (*P* = 0.031). As shown in Table 1, in the first quartile (35–54 years), 88.0% were male, whereas in the fourth quartile (69–87 years), 52.8% were male. There were no statistically significant differences in smoking history (*P* = 0.098), alcohol consumption (*P* = 0.518), diabetes (*P* = 0.082), hypertension (*P* = 0.287), ischemic cerebral infarction history (*P* = 0.376), atrial fibrillation (*P* = 0.171), or lipid metabolism disorders (*P* = 0.669) across age groups. Similarly, no significant differences were found in BMI, NIHSS score at admission, systolic blood pressure (SBP), or diastolic blood pressure (DBP) among the age quartiles. Additionally, no statistically significant differences in TOAST classification were observed across the age quartiles (*P* = 0.055).

**Table 1:** Baseline characteristics of stroke patients by age quartile.

**Characteristic**	**Age quartile**				
	**Quartile 1 (*****n** =* **25, 35–54)**	**Quartile 2 (*****n** =* **30, 55–63)**	**Quartile 3 (*****n** =* **30, 64–68)**	**Quartile 4 (*****n** =* **36, 69–87)**	***P*** **-value**
**Gender (%)**					0.031^*^
Male	22 (88.000%)	21 (70.000%)	18 (60.000%)	19 (52.778%)	
Female	3 (12.000%)	9 (30.000%)	12 (40.000%)	17 (47.222%)	
**Smoking history (%)**					0.098
Yes	8 (32.000%)	3 (10.000%)	7 (23.333%)	4 (11.111%)	
No	17 (68.000%)	27 (90.000%)	23 (76.667%)	32 (88.889%)	
**Alcohol history (%)**					0.518
Yes	7 (28.000%)	5 (16.667%)	7 (23.333%)	5 (13.889%)	
No	18 (72.000%)	25 (83.333%)	23 (76.667%)	31 (86.111%)	
**Diabetes (%)**					0.082
Yes	2 (8.000%)	7 (23.333%)	11 (36.667%)	7 (19.444%)	
No	23 (92.000%)	23 (76.667%)	19 (63.333%)	29 (80.556%)	
**Hypertension (%)**					0.287
Yes	8 (32.000%)	17 (56.667%)	13 (43.333%)	14 (38.889%)	
No	17 (68.000%)	13 (43.333%)	17 (56.667%)	22 (61.111%)	
**Ischemic cerebral infarction history (%)**					0.376
Yes	1 (4.000%)	5 (16.667%)	2 (6.667%)	3 (8.333%)	
No	24 (96.000%)	25 (83.333%)	28 (93.333%)	33 (91.667%)	
**Atrial fibrillation (%)**					0.171
Yes	0 (0.000%)	1 (3.333%)	4 (13.333%)	2 (5.556%)	
No	25 (100.000%)	29 (96.667%)	26 (86.667%)	34 (94.444%)	
**Lipid metabolism disorder (%)**					0.669
Yes	3 (12.000%)	2 (6.667%)	1 (3.333%)	3 (8.333%)	
No	22 (88.000%)	28 (93.333%)	29 (96.667%)	33 (91.667%)	
Body mass index (kg/m^2^)	23.877 ± 1.503	24.609 ± 1.471	24.594 ± 2.609	24.431 ± 1.714	0.458
NIHSS (Day 1)	2.400 ± 1.528	2.067 ± 1.311	2.333 ± 1.213	2.639 ± 1.496	0.427
SBP (mmHg)	134.360 ± 14.980	139.367 ± 14.845	141.967 ± 19.038	137.833 ± 20.907	0.465
DBP (mmHg)	81.520 ± 10.038	80.533 ± 8.713	80.933 ± 9.251	78.056 ± 9.027	0.448
TMAO (μmol/L)	92.054 ± 42.259	219.081 ±232.905	170.245 ±170.466	179.829 ± 125.722	0.036^*^
TML (μmol/L)	75.134 ± 43.457	70.648 ± 25.896	67.145 ± 25.257	68.005 ± 22.639	0.748
PAGln (μmol/L)	186.867 ± 95.493	222.472 ±119.712	276.454 ±180.942	433.108 ±474.033	0.004^**^
**TOAST classification**					0.055
LAA	4 (16.000%)	9 (30.000%)	7 (23.333%)	16 (44.444%)	
CE	0 (0.000%)	1 (3.333%)	3 (10.000%)	2 (5.556%)	
SAA	20 (80.000%)	20 (66.667%)	19 (63.333%)	14 (38.889%)	
SUE	1 (4.000%)	0 (0.000%)	1 (3.333%)	4 (11.111%)	

Values are presented as mean ± standard deviation (SD) or n (%). *P*-values were calculated using the Kruskal-Wallis test for continuous variables and the Chi-square test or Fisher's exact test for categorical variables (where expected cell count < 10). Significance levels are marked as: ^*^p < 0.05, ^**^p < 0.01, ^***^p < 0.001. TOAST Classification includes: LAA, large artery atherosclerosis; CE, cardioembolism; SAA, small artery occlusion; SUE, stroke of undetermined etiology.

Significant differences in metabolite levels were observed across age groups. Table 1 shows that TMAO levels differed significantly (*P* = 0.036), with the first quartile having a mean level of 92.05 ± 42.26 μmol/L, which increased to 219.08 ± 232.91 μmol/L in the second quartile. PAGln levels also varied significantly among age groups (*P* = 0.004), increasing from 186.87 ± 95.49 μmol/L in the first quartile to 433.11 ± 474.03 μmol/L in the fourth quartile.

### Non-linear relationship between age and PAGln levels

**Figure 1:**
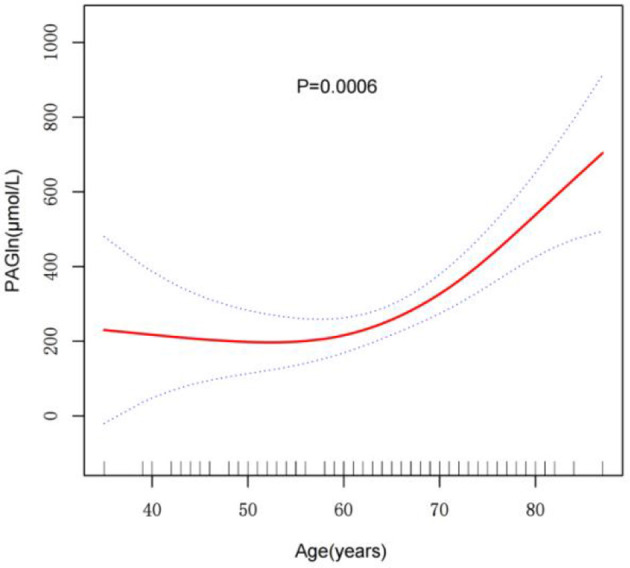
Smooth curve fitting of the relationship between age and PAGln levels. The relationship was modeled using a GAM adjusted for sGender, Alcohol, Smoking, BMI, Ischemic Cerebral Infarction History, Atrial Fibrillation, Hypertension, Diabetes, Lipid Metabolism Disorder, TOAST Classification, and NIHSS Score (Day 1). The red line indicates the smoothed trend of PAGln levels with age, and the blue dashed lines represent 95% confidence intervals. A significant non-linear association was observed (*P* = 0.0006***). ***p < 0.001.

The non-linear relationship between age and PAGln levels was assessed using GAMs. After adjusting for multiple confounders, including gender, smoking, alcohol consumption, BMI, ischemic cerebral infarction history, atrial fibrillation, hypertension, diabetes, lipid metabolism disorders, TOAST classification, and NIHSS score, a significant non-linear association between age and PAGln levels was observed (*P* = 0.0006) (Figure 1). The fitted smooth curve indicated that PAGln levels followed a significant non-linear “exponential” growth pattern with increasing age.

A segmented regression model identified a threshold point at 71 years for the relationship between age and PAGln levels (Table 2). Below 71 years, the effect of age on PAGln levels was not statistically significant, with an effect size of 2.135 (95% CI: −4.659, 8.929, *P* = 0.5394). In contrast, above 71 years, the effect size significantly increased to 35.520 (95% CI: 19.042, 51.998, *P* < 0.0001), indicating a marked exponential rise in PAGln levels. The significance of this threshold effect was further validated by a likelihood ratio test (*P* < 0.001), confirming a highly significant non-linear association between age and PAGln levels after 71 years, with a rapid increase in this age range.

**Table 2:** Threshold effect analysis of age and PAGln levels.

**Age**	**PAGln adjusted β (95% CI), *p* value**
Model I	9.103 (3.429, 14.777) 0.0022^**^
**Model II**	
Breakpoint (K)	71
β1 (< K)	2.135 (-4.659, 8.929) 0.5394
β2 (> K)	35.520 (19.042, 51.998) < 0.0001^***^
β2/β1	33.385 (13.717, 53.054) 0.0012^**^
Logarithmic likelihood ratio test *p*-value	< 0.001^***^

Values are presented as Adjusted β(95% CI) with *p*-values. *P*-values were calculated using a segmented regression model with a threshold effect analysis. The model is adjusted for the following confounders: gender, alcohol consumption, smoking history, BMI, ischemic cerebral infarction history, atrial fibrillation, hypertension, diabetes, lipid metabolism disorder, TOAST classification, and NIHSS score (Day 1). Significance levels are marked as: ^*^p < 0.05, ^**^p < 0.01, ^***^p < 0.001. The logarithmic likelihood ratio test confirmed the significance of the threshold effect.

### Subgroup analysis

A subgroup analysis was performed to assess the stability of the association between age and PAGln levels across different population characteristics. The results showed that this association remained consistent regardless of population characteristics. As presented in Table 3, none of the stratification variables—including NIHSS score, gender, TOAST classification, smoking, alcohol consumption, diabetes, hypertension, ischemic cerebral infarction history, atrial fibrillation, or lipid metabolism disorders—significantly affected the relationship between age and PAGln levels. All interaction *P*-values were >0.05, indicating that none of the covariates significantly modified the effect of age on PAGln levels.

**Table 3:** Subgroup interaction analysis of age and PAGln levels.

**Subgroups**	**N**	**β (95% CI), *p* value**	***p* for interaction**
**NIHSS score quartile**			0.2791
0–1	31	6.563 (−3.106, 16.232) 0.1863	
2–2	35	4.851 (−4.652, 14.353) 0.3194	
3–5	55	12.769 (4.676, 20.862) 0.0026	
**Gender (%)**			0.1192
Male	80	6.734 (0.222, 13.246) 0.0453	
Female	41	14.722 (5.175, 24.268) 0.0032	
**TOAST classification**			0.8263
LAA	36	5.844 (−3.308, 14.997) 0.2145	
CE	6	85.008 (21.738, 148.277) 0.0102	
SAA	73	4.863 (−1.220, 10.945) 0.1212	
SUE	6	−51.501 (−130.308, 27.306) 0.2041	
**Smoking history (%)**			0.0745
Yes	22	−0.436 (−13.150, 12.277) 0.9465	
No	99	10.604 (4.697, 16.510) 0.0007	
**Alcohol history (%)**			0.1559
Yes	24	2.610 (−8.684, 13.903) 0.6516	
No	97	10.829 (4.605, 17.052) 0.0009	
**Diabetes (%)**			0.3736
Yes	27	3.372 (−11.548, 18.292) 0.6587	
No	94	9.739 (3.853, 15.626) 0.0016	
**Hypertension (%)**			0.6627
Yes	52	10.418 (1.801, 19.035) 0.0197	
No	69	8.405 (1.753, 15.057) 0.0149	
**Ischemic cerebral infarction history (%)**			0.2031
Yes	11	40.487 (−17.459, 98.433) 0.1741	
No	110	7.537 (1.593, 13.480) 0.0147	
**Atrial fibrillation (%)**			1.0000
Yes	7	73.249 (−453.030, 599.528) 0.7856	
No	114	4.593 (−0.253, 9.439) 0.0663	
**Lipid metabolism disorder (%)**			0.2912
Yes	9	3.172 (−10.127, 16.471) 0.6412	
No	112	10.051 (4.058, 16.044) 0.0014	

Values are presented as β(95% CI) with *p-*values. *P*-values were calculated using regression analysis with interaction terms for each subgroup. The model adjusts for potential confounders including age, gender, smoking history, alcohol consumption, BMI, ischemic cerebral infarction history, atrial fibrillation, hypertension, diabetes, lipid metabolism disorder, and TOAST classification. Interaction *p-*values test the effect modification between age and PAGln levels across various subgroups.

## Discussion

This study represents a significant advancement by revealing a non-linear exponential relationship between age and the gut microbiota-derived metabolite PAGln in stroke patients, especially noting a marked increase after the age of 71. These findings offer new insights into metabolic changes in stroke patients, with important implications for future clinical management, particularly for elderly populations.

Our results indicate a notable increase in PAGln levels in patients aged 71 and above, suggesting significant changes in gut microbiota function and host physiology. Previous research has shown that aging is associated with reduced gut microbiota diversity and an increase in pathogenic bacteria, which may contribute to elevated PAGln production (13, 14). Yu et al. also reported a significant association between higher PAGln levels and increased white matter hyperintensities (WMH) burden, particularly in patients with moderate-severe WMH (15). This finding supports the potential role of PAGln as a biomarker for assessing metabolic dysfunction in stroke patients. GAMs analysis identified 71 years as a crucial threshold for significant changes in PAGln levels, indicating a point of potential metabolic dysregulation. Clinically, this threshold reflects an increased metabolic burden in older adults, which could elevate the risk of thrombosis and stroke-related complications.

This study emphasizes the effects of age on PAGln metabolism. Previous research has associated elevated PAGln levels with an increased risk of thrombosis, particularly in cardiovascular diseases (16, 17). Our study specifically examined age-related differences in PAGln levels among stroke patients. PAGln is known to enhance platelet reactivity, thereby increasing the risk of thrombosis, which is particularly concerning for elderly stroke survivors (18, 19). The marked rise in PAGln levels in patients over 71 suggests its potential use as a biomarker for assessing metabolic burden and thrombosis risk, providing valuable insights for individualized risk assessment. These findings point to clinical applications, such as monitoring and regulating PAGln levels in elderly patients to reduce thrombosis risk and improve stroke recovery.

PAGln levels display a non-linear growth pattern, reflecting the complexity of its age-dependent metabolic regulation. Before the age of 71, PAGln levels remain relatively stable, possibly due to effective metabolic stress regulation in younger and middle-aged individuals. With aging, reduced gut barrier function and increased intestinal permeability may lead to elevated PAGln production (20–22), which could explain the sharp rise observed after age 71. Our threshold effect analysis confirms this point as a critical turning point, beyond which PAGln levels rise significantly, indicating metabolic dysregulation and heightened thrombosis risk.

The subgroup analysis showed that PAGln levels increased exponentially in individuals aged over 71, irrespective of gender, smoking history, or alcohol consumption. These findings emphasize that age is the primary factor influencing PAGln metabolism, while other individual characteristics contribute minimally. Even after adjusting for multiple confounding factors, the rising trend in PAGln levels remained significant, indicating an independent association between age and PAGln levels. This highlights the potential of PAGln as a biomarker for assessing metabolic burden in elderly stroke patients, particularly in the absence of other notable risk factors, thereby enabling more targeted and individualized patient management. PAGln levels could thus serve as a key biomarker for predicting stroke recurrence, allowing for more personalized management approaches.

This study provides significant insights by revealing a non-linear exponential relationship between PAGln levels and age, particularly with a marked increase after age 71. These findings have important implications for clinical practice. PAGln could serve as a valuable biomarker for metabolic monitoring in stroke patients, especially among the elderly, providing early indications of metabolic dysfunction and thrombosis risk. Furthermore, patients with significantly elevated PAGln levels—particularly those over the age of 71-may benefit from personalized interventions, including dietary changes, modulation of gut microbiota, or pharmaceutical therapies to reduce metabolic burden and thrombosis risk.

### Limitations

This study has several limitations. First, the sample size of 121 ischemic stroke patients is relatively small, which may affect the generalizability of the findings. Second, it is a single-center study, which may introduce selection bias. Third, although we adjusted for several confounders, factors such as dietary habits and gut microbiota composition were not considered, which could influence PAGln levels. Furthermore, the cross-sectional design of this study limits the ability to infer causality between age, PAGln levels, and thrombosis risk. Finally, the lack of long-term follow-up data means that we cannot determine whether elevated PAGln levels persist over time or whether they contribute to long-term stroke outcomes. These limitations highlight the need for future studies with larger sample sizes, multi-center data, and longitudinal follow-up to confirm our findings and explore the potential causal relationships between PAGln levels, stroke prognosis, and metabolic dysfunction.

## Conclusion

This research offers new insights into the metabolic characteristics of elderly stroke patients by revealing a non-linear exponential relationship between PAGln levels and age. Specifically, the sharp increase in PAGln levels observed after the age of 71 suggests a heightened risk of thrombosis and stroke in this population. Thus, monitoring PAGln levels could provide valuable evidence for personalized management and early intervention strategies in elderly patients.

## Data Availability

The original contributions presented in the study are included in the article/Supplementary material, further inquiries can be directed to the corresponding authors.
