# Supplementary material for: Threshold of phenylacetylglutamine changes: exponential growth between age and gut microbiota in stroke patients
Source: Front Neurol. 2025 May 21;16:1576777. doi: 10.3389/fneur.2025.1576777 (PMC12133545; doi:10.3389/fneur.2025.1576777)

**Table S1** - Comparison of PAGln Levels Across TOAST Stroke Subtypes

| **TOAST Classification** | **LAA**  **(n=36)** | **CE**  **(n=6)** | **SAA**  **(n=73)** | **SUE**  **(n=6)** | **P value** |
| --- | --- | --- | --- | --- | --- |
| PAGln (µmol/L) | 422.50 ± 340.09 | 506.03 ± 825.03 | 215.96 ± 153.30 | 203.44 ± 136.59 | <0.001 |

Notes:TOAST Classification: Large Artery Atherosclerosis (LAA)、Cardioembolism (CE)、Small Artery Occlusion (SAA)、Stroke of Other Determined Etiology (SOE)、Stroke of Undetermined Etiology (SUE).

**Table S2** - Association Between PAGln Levels and the Occurrence of LAA Versus SAA

| **Exposure** | **Model** | **OR (95% CI)** | **P-value** |
| --- | --- | --- | --- |
| PAGln (µmol/L) | Adjust I | 1.004 (1.001, 1.007) | Reference |
| PAGln (µmol/L) | Adjust II | 1.009 (1.004, 1.013) | 0.0004 |

Note: Adjust I model was adjusted for age, sex, BMI, smoking, and alcohol consumption.Adjust II model was further adjusted for ischemic cerebral infarction history, atrial fibrillation, hypertension, diabetes, and lipid metabolism disorders.

**Table S3** - Comparison of PAGln Levels Between Patients With and Without Intravenous Thrombolysis

| **Thrombolysis** | **No (n=58)** | **Yes (n=63)** | **Standardized Difference** | **P-value** |
| --- | --- | --- | --- | --- |
| PAGln (µmol/L) | 300.97 ± 285.98 | 282.15 ± 309.05 | 0.063 (–0.294, 0.420) | 0.729 |

**Table S4** - Threshold Effect Analysis of Age and PAGln Levels

| **Age** | **PAGln Adjusted β (95% CI), p value** |
| --- | --- |
| Model I | 9.103 (3.429, 14.777) 0.0022** |
| Model II |  |
| Breakpoint (K) | 71 |
| β1 (< K) | 2.135 (-4.659, 8.929) 0.5394 |
| β2 (> K) | 35.520 (19.042, 51.998) <0.0001*** |
| β2/β1 | 33.385 (13.717, 53.054) 0.0012** |
| Logarithmic likelihood ratio test p-value | <0.001*** |

**Notes:** Values are presented as Adjustedβ(95% CI) with p-values.P-values were calculated using a segmented regression model with a threshold effect analysis. The model is adjusted for the following confounders: gender, alcohol consumption, smoking history, BMI, ischemic cerebral infarction history, atrial fibrillation, hypertension, diabetes, lipid metabolism disorder, TOAST classification, and NIHSS score (Day 1).

**Significance levels are marked as:** *p<0.05, **p<0.01, **p<0.001.

The logarithmic likelihood ratio test confirmed the significance of the threshold effect

**Figure S1** - CONSORT Flow Diagram of Patient Enrollment and Analysis


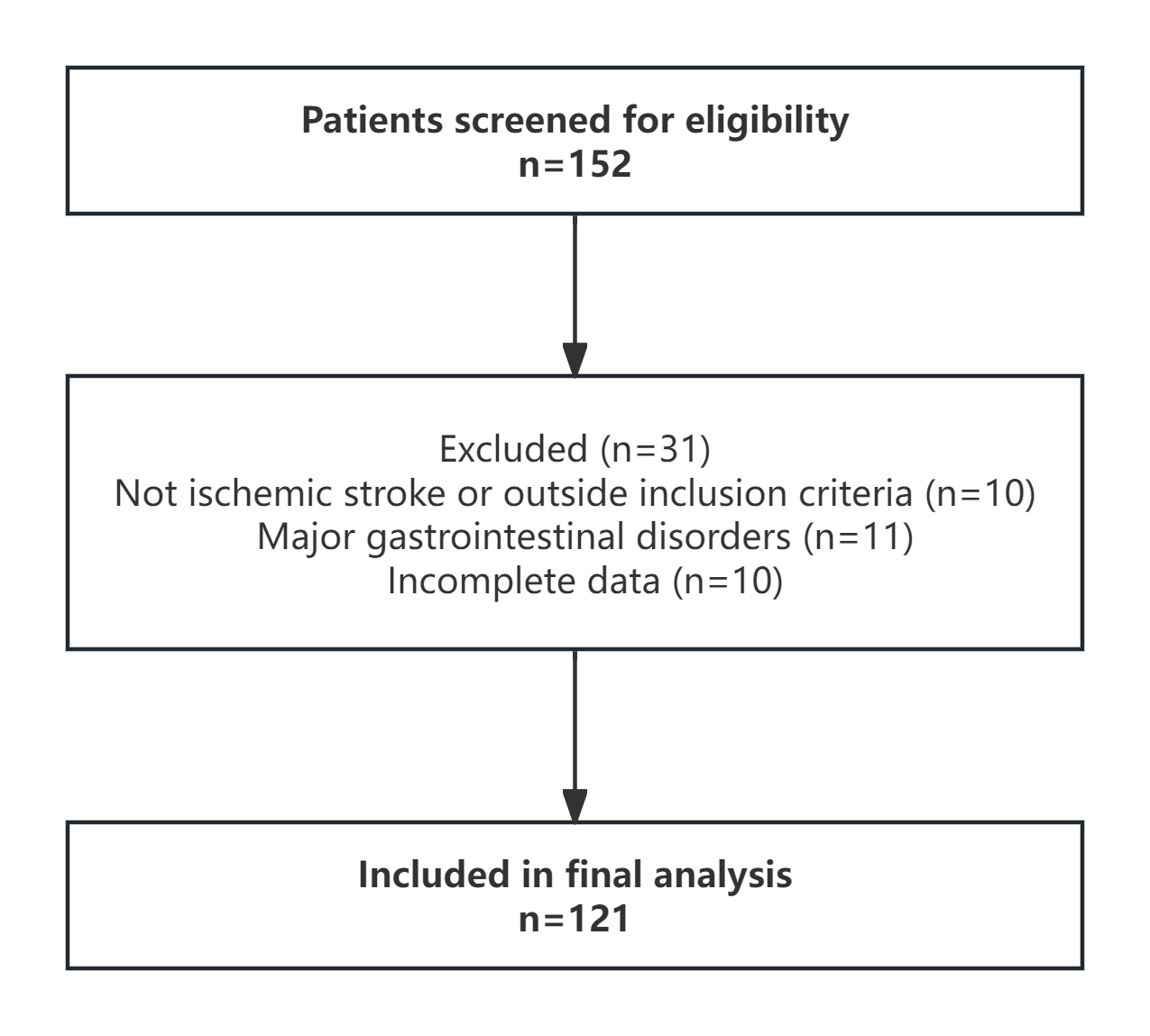

Supplement: Supplementary file 1 [file Supplementary_file_1.docx]
